# Foreign

**Published:** 1840-11-14

**Authors:** 


### FOREIGN.

*On the Contractility of the Blood-vessels.* By Dr. Henle, of Berlin.—The object of the author in the present paper is to offer anatomical evidence in favour of the views respecting the nature of inflammation, which he expressed in his "Pathological Investigations," and of which we gave a full analysis in our Number for April last. He now, in support of those opinions, advances that the structure of the middle coat of the artery is intermediate between cellular and muscular tissue; and that the vessels must therefore be liable to those changes of caliber under the direct or sympathetic influence of the nerves to which he believes that inflammation is due. With reference to his conclusions, we can only repeat the opinion given in the review already mentioned, that mere dilatation of blood-vessels is not inflammation, and that even if it were proved that depression of the supposed motor nerves of the blood-vessels could result as an antagonist phenomenon of the excitement of the sensitive nerves, this would constitute but one, and not the most important part of inflammation. However, the present anatomical observations of the author are not much the less interesting because he makes them the groundwork of incorrect pathology; if true, they afford additional proof, and indeed the only evidence that was wanting, to establish that the blood-vessels are subject to changes of their caliber under other than mere physical influences. And it is but fair that this evidence should be supplied from the school of Müller, who has done so much to throw discredit on the opinions of those who regarded the fact as already reasonably demonstrated.

The microscopic elements of the middle coat of the artery are broad and very flat slightly granulated fibres or bands, which lie in rings around the internal membrane. In the innermost layers they readily break up into tolerably similar long rhombic lamellæ, which are much like flattened cuticle cells, but are longer in proportion to their breadth. Of these lamellæ some are quite homogeneous, others present at one part a dark oval spot of the form of the common nuclei of cells, which lies with its greatest diameter in the long axis of the lamella; and, lastly, on others this spot is drawn out into a longer and fine dark streak which often extends over the whole lamella, but often over only a part of it, and often is replaced by a row of small dark points.

In the more external layers the lamellæ unite into long, and very seldom, if ever, branched fibres; the dark streaks also become connected together, not only joining with one another by their ends, but at the same time sending off lateral branches, by which they anastomose. Thus the middle coat of the artery consists of numerous layers of granulated transverse bands (of 0.003 lines diameter,) and of a system or network of dark streaks among them. These have some resemblance to elastic fibres, and have given rise to the opinion that this coat consists of elastic tissue. But a true elastic coat exists only on the larger arteries external to the middle one. The granulated bands of the middle coat dissolve in acetic acid, the dark branched streaks do not, and they may therefore by its means be easily demonstrated.

In the larger veins there is an exactly similar layer of transverse fibres next to the inner coat; but it is always very thin, and may be entirely absent. (In the inner coat of the veins, however, a longitudinal layer of such fibres, which is much thinner or absent in the arteries, is in general considerably developed.) If one follows these fibres outwards it is seen how, though at first they were straight and stiff, they gradually assume the peculiar waviness of fasciculi of cellular tissue, and how at last there appears in them an obscure and then a more and more evident breaking up into separate parallel and tortuous filaments; for each granulated flat fibre there is a fasciculus of cellular tissue. The dark streaks on these fasciculi at first form a network like that which they present on the granulated fibres of the middle arterial coat; gradually they become finer and more translucent, the lateral branches fade, and nothing is seen but dark and very large wavy fibres, such as universally occur between the fasciculi of cellular tissue. These fasciculi become pale and dissolve in acetic acid; the fibres remain.

If the muscular coat of the stomach or of the intestine be examined successively from the serous to the mucous membrane, there are found externally lamellæ similar to those in the innermost layers of the middle arterial coat, with the same nuclei and the same development of the nuclei into streaks. Towards the mucous membrane, the laminæ coalesce into broad fibres, the well-known organic muscular fibres which in their microscopic and chemical characters agree with the fibres of the middle coat of the artery, but which frequently exhibit obscure divisions into finer parallel fibres. The dark streaks are much finer, fewer, less branched, and more similar to the fine interstitial fibres of the cellular fasciculi just mentioned. The so-called muscular *fibres* of the stomach and intestines then correspond to the *fasciculi* of the cellular tissue and of the striated muscles.

I have not yet, says Dr. Henle, traced the transition from the organic muscular fibres to the striated muscular fasciculi, and I shall merely mention that in the latter also the system of dark interstitial fibres insoluble in acetic acid occurs sometimes in the form of nuclei, sometimes of longer streaks, and sometimes of extremely fine wavy fibres. It is well known that the organic and striated muscular fasciculi dissolve in acetic acid. The chemical difference between the middle coat of the artery and the muscles is explained by the predominant quantity of interstitial fibres in the former. The anatomical identity of the above-mentioned tissues in all essential points is thus, I believe, demonstrated: with the less essential anatomical differences there correspond differences of functions. The striated muscles are generally subject to the will, and react on mechanical and electric stimuli, but not on the application of cold; the heart is an exception, having striated fasciculi, but not being voluntarily moveable; the organic muscles react as the striated do, but are removed from the influence of the will; the middle coat of the artery is anatomically similar to the organic muscular membrane, it is, like them, involuntary, it reacts, like them, on mechanical stimuli, and also on the application of cold, but not of electricity; the contractile cellular tissues, for example, the tunica dartos, similarly involuntary, has the same relations to cold as the middle coat of the artery, but is not excited to contraction by mechanical impressions. In the animal muscles, contraction following upon irritation is sudden and single; in the heart sudden and peristaltic; in the intestine slow and peristaltic; in the arteries and the cellular tissue slow and continuous.

The contractility of the larger arteries is proved by experiment, and we may venture to ascribe it to the smaller ones as far as they agree in structure with the larger, that is, so far as the layer of circular fibres is continued. Now this may be recognized on vessels of from 0.015 to 0.02 of a line in diameter, by rendering them transparent with acetic acid, and examining them under the microscope. The interstitial dark streaks are then seen running straight, or somewhat obliquely, round the vessel, at distances from each other which are equal to the breadth of a granulated fibre, in single layers on the small, and in numerous layers on the large vessels. Separate fibres may also be exhibited on arteries of this size by tearing them. In smaller arteries, however, this is not possible; but even on those of 0.007 of a line in diameter (including their walls) there may be seen over the epithelium a layer of transverse, oval, and in part very elongated nuclei, which may be recognized as the origins of the dark interstitial fibres of the middle coat, by the circumstance that there is a gradual transition to the form of the latter, and that they already lie at the same distance from one another. Between them, therefore, the same substance must exist as between the interstitial fibres of the middle coat of the larger arteries, though it is not yet arranged in separate circular fibres. In vessels of this degree of minuteness the distinctions between arteries and veins are no longer to be discerned. Capillaries of less than 0.007 diameter (the smallest are from 0.002 to 0.003) have indeed separate transverse oval nuclei, but they have no special middle coat. The smallest consist only of a thin quite structureless membrane, in which oval nuclei lie here and there in a longitudinal position: those that are something larger have an epithelium of nuclei within this structureless primary membrane.

For the opinion that the contraction of the membrane of the vessels, like that of the muscles, is under the influence of the nerves, I can adduce an anatomical fact. Purkinje has seen fine nervous twigs on the cerebral vessels of sheep; and Valentin both on them and on many other blood-vessels; and I have myself often observed nervous filaments presenting the character of the so-called organic fibres on small vessels, such as one can see undissected with powerful lenses. On a vessel of the pia mater of one-fifth of a line in diameter, I saw such a nervous faciculus (of 0.009 of a line in diameter) ascending to the upper wall of the vessel, which was turned towards the eye, then turning round it to its posterior wall, and then continuing its course in the same direction. I have seen this winding of the nerves round the vessels only on very small portions of the latter, but I have observed it so often that I cannot regard it as an accident. In one case a smaller fasciculus was given off; it consisted of only three or four fibres and passed along the vessel. —*Brit. and For. Med. Rev. from Casper's Wochenshrift. Mai* 23, 1840.

---

*On the Alterations which Nervous Fibres undergo after Division.* By Professor NASSE, of Marburg.—The results of the lengthened investigations which form the subject of this paper may be briefly stated.

In frogs, the natural average thickness of the filaments of the ischiatic nerve is 0.000416 inch; the majority are much smaller: the filaments of the posterior tibial are of about the same size; those of the brachial rather less, viz., 0.0003835. The smallest size observed was 0.00027 or 0.00028, and this was so common that the author is inclined to believe it to be the true diameter of all filaments unaltered by external influences, or at least of one kind of them. The limits of size were 0.00037 and 0.00045.

The filaments above the division, after it has healed (and usually about five months after the operation,) are almost constantly larger than those below it. The average increase of size is 0.00005 to 0.00006.

The average size of the filaments which are paralyzed by the division, is but little different from that of health; many months after the operation they are found only a little smaller. Their decrease in size is greatest when the animal is generally much emaciated; and is greatest of all when the main artery of the limb is divided at the same time as the nerve.

After destroying the connexion of a nerve with the spinal cord, its filaments first lose their cylindrical appearance, and from wrinkling acquire a transversely striated appearance, so that they seem to be divided iuto a number of short pieces. Next, small fat globules form from the breaking-up of the medulla, and the filaments become darker and more opaque. Afterwards these fat globules unite into larger drops, and then the walls of the nervous tubuli gradually diminish in their size.

The filaments that form in the substance by which the divided extremities are united are rather smaller than the old ones, but in all respects similar to nervous fibres; their average diameter is 0.000374.

Similar observations made on rabbits had very nearly the same result; but it was remarked that as in warm-blooded animals generally the nutrition of all the tissues is more under the influence of the spinal cord than in the cold-blooded, so here the destruction of the divided nerves was much more rapid in rabbits than in frogs; and that although kept without food for five months in the most unfavourable season of the year, the latter gave evidence of much more energy of reparation than the former which were well fed.

Although there was no doubt of new nervous fibres being formed between the extremities of the divided nerves, yet Nasse never found the motion or sensation return in the paralyzed limbs, though he sometimes kept the animal for three quarters of a year. He imagines that in the cases in which others have seen such a restoration of power, the divided portions must have been at once placed in exact apposition, so that the filaments could not lose their oily contents; for he clearly determined that the least alteration in the structure of a filament is sufficient to destroy its conducting power.—*Ibid. from Müller's Archiv.* 1839. Heft v. p. 405.

*Practical Observations on the Diseases of Peru, described as they occur on the Coast and in the Sierra.* By Archibald Smith, M. D.

(Continued.)

I.—Diseases of the Coast continued.

*Diseases of the Abdominal Cavity—Fatiga.*—I cannot enter upon an account of the various diseases to which the abdominal viscera are more particularly liable on the Coast of Peru, without trying to give the reader some idea of what is meant by *Fatiga.*

This expression is wide in its acceptation, and often used to signify a general feeling of uneasiness which attends certain disorders not referable by positive pain to any particular organ.

It is a common symptom during the hot stage of ague, and various disorders of the viscera, and seems to proceed from disturbance of the ganglionic system of nerves. There is hardly a female patient in Lima suffering under any disorder of the digestive organs but enumerates this sensation among the symptoms of her ailment.

Hearing *fatiga* so often mentioned by the sick, I endeavoured to ascertain what precise meaning was attached to it in particular cases. Some signify by the expression a feeling of oppression about the præcordia, accompanied with anxiety. Others say, that they mean by *fatiga* a peculiar sort of tightness at the chest, with shortness of breathing, like that which is experienced on stooping, running, or hurriedly ascending stairs. It is used very commonly, especially by ladies, to express a feeling of sinking, or of vacuity at the stomach, often relieved by a cup of chicken-soup or chocolate. It is also employed to denote a sense of weight and weariness referred to the region of the stomach, attended with loathing of food, consequent, perhaps, on an old hepatic disorder, originally caused by the inward working of suppressed anger. This species of *fatiga* is said to be relieved, from time to time, by a few drops of laudanum.

It is a symptom of common dyspepsia, more or less aggravated when the stomach is distended with flatus. In the latter instance, the female patient imagines that she experiences what is vulgarly called *a levantarse le madre*, or a rising of the womb, which produces the *fatiga*,—in other words, that it is an attack of hysterical globus.

*Dyspepsia.*—The process of digestion is usually very slow with the natives of the coast; and when in any degree indisposed, they are commonly cautious not to load the stomach afresh, whilst yet affected with flatulency or eructations, which is not unfrequently the case, for seven or eight hours after an ordinary meal. Indeed one of the commonest signs of dyspepsia is that of which the natives so often complain, *eructando la comida hasta tarde*, viz. being troubled with eructation to a late hour after food. And among the most obvious causes of this affection is the general practice of using the cigar, and so creating an excessive secretion and ejection of the saliva, necessary for a healthy digestion.

A distinguished old Spaniard, for more than half a century resident in Lima, used to give the following rule for the enjoyment of health and longevity in that place. "To breakfast lightly, dine heartily, sup sparingly, and never break the night's rest."* This indeed may be a very good summary for Lima, where the people are generally cheerful and fond of amusement and sauntering,—circumstances which Cicero did not overlook when he inculcated that health required "concoctionem, vitationem lassitudinis, ambulationem moderatam, oblectationem, facilitatem alvi."

It is a prevalent belief in Lima, that men of letters in that climate are capable of very little literary labour without soon disturbing the organs of digestion. I once attended a person of spare form and pale countenance, who was habitually subject to dyspepsia. The disorder seemed to have been occasioned by a sedentary life, pensive disposition, and much dedica-

* His own words are "Almorzar poco; comer quanto queres; cenar lo menos que puedes; y no pasar mala noche."

tion to reading. His feet were usually cold, and his digestion irregular, as was indicated by the varying quality of his stools, acid eructations, and flatulent movements in the bowels. If at any time he drank iced-water at dinner, or took a glass of cold water before the process of digestion in the stomach and duodenum was finished, that is, before five or six hours had elapsed after last meal, his bowels were sure to be disturbed in consequence; his head became heavy, and he felt drowsy. To avoid this disagreeable sense of weight and fulness, which gave him the idea of a rush of blood to the brain, he left off cold water entirely, and used with benefit tepid water for his ordinary drink.

I have known other dyspeptics of delicate constitution affected very much in the same manner. In this case, the affection of the head was more prominent than ordinary in those other instances in which we find that the general symptoms, and the effects of cold drink, are nearly the same.

Though it is a prevalent custom to drink iced-water at meals during the summer months, many delicate dyspeptics, like the individual just alluded to, cannot use it freely without suffering griping and other disturbances in the bowels, for which reason they prefer tepid water for common drink. With others, however, of a different fibre and temperament, cold drinks agree remarkably well during the warm weather, when the heat and irritability of the stomach are evidently greatest; and indeed many of them cannot digest well without the aid of ice.

It is a curious fact, connected with the local custom of using ice, that patients are met with whose powers of digestion are so feeble that the simplest chicken-soup or chicken-tea would sour on the stomach, unless this effect were prevented by cooling beverages. In some cases of chronic diarrhœa and dysentery, I have observed that the chicken-tea occasioned frequent and distressing calls to stool, when not restrained by the use of ice. This valuable remedy seems to impart tone or power, by lowering the morbid irritabilitity of the stomach. From the Lima chalanes or jockeys, the new comer to a warm climate may take a lesson. They are remarkable for observing the different effects of cold and warm weather on the cattle under their care. In the cold and damp months they give their mules and horses grain with advantage; but in the heat of summer they withhold corn. They observe that grain, during the warm season on the coast, brings on a cutaneous eruption difficult to cure; and which is only removed by change of climate, or sending the animal to grass.

In illustration of the good effects on the human constitution of exchanging a stimulating mode of living, such as is fitted for a cold northern climate, for a more temperate regimen, better suited to a tropical one, I would adduce the following case.

An European in the prime of life, of a florid complexion, vigorous frame, and sanguine temperament, had once or twice suffered from acute hepatitis and bilious remittent fever; but, when in health, he never left off his wine, and was greatly addicted to strong coffee. Having so long indulged in these heating beverages, as he thought with sufficient moderation, because with perfect sobriety, he could not be persuaded by his family, which was Limenian, that the use of these articles could have any share in keeping up the habitual dyspeptic symptoms which he suffered. This affection was characterized by much flatulency, with such an inflated state of the belly, as often rendered his widest vest too tight for him. He had three loose stools daily. At length he was prevailed upon to make trial of a different regimen. He took no more hot suppers; he left off coffee and chocolate at night, and confined himself to a glass of iced-water before going to bed. At dinner, instead of his customary allowance of wine, he only drank a little Bordeaux, mixed with a plenty of water. The consequence of this new plan was, a cheerful mind, an easy stomach, and perfect health, with only one natural stool daily. I believe that this person's example might be often followed with no small advantage.

It may be remarked, that the beer of the country, which is made from Indian corn, and without the addition of hops, or bitter of any kind, sometimes procures a good digestion, when foreign beer or porter would be sure to have the contrary effect, and to render one bilious.

It is only among the dark races that spirituous liquors are much used; for, except in small quantities taken as drams at meals, they are, under all ordinary circumstances, considered to be contraindicated by the general influence of the climate. A spirituous purgative, for example, tincture of senna or rhubarb, usually irritates the stomach so much as to render it mostly always an improper remedy. Notwithstanding this peculiarity of operation in certain stimulants, others appear to be borne with comparative ease; for the stomach is daily excited by the excessive use of agi, (pepper,) with little if any apparent inconvenience. But to this condiment, acting as a local cause, it may partly be owing that the papillæ of the tongue, especially near the root of the organ, are very often greatly elevated and enlarged in the natives.

An exceedingly irritable state of the stomach is sometimes discovered in delicate persons, without being accompanied with any unusual redness or morbid appearance of the tongue, though the gums may be spongy and ready to bleed on slight cause.

In many persons cutaneous efflorescences, or red blotches attended with troublesome itching, frequently manifest themselves when the ingesta happen to disagree with the stomach, or irritate the intestinal mucous membrane.

I have observed an instance wherein, after many months of continued relapses in case of quotidian or tertian ague, the irritability of stomach gradually increased until the utmost rigor of diet was indispensable. And after a plain and simple meal, though no pain or heat should be felt in the stomach, to which the stimulus was directly applied, the nose became red and hot, and the facial artery pulsated with increased fulness and strength.

A wine-glassful of undiluted wine would produce a similar effect. A teaspoonful of spirits could not be taken without giving rise to a disagreeable sense of heat at the throat and stomach. Epsom salts, dissolved in the infusion of cinchona, would sit easily, and act efficiently on the bowels, when other common evacuants caused increased irritation. No rich gelatinous soups, sweatmeats, or fresh pork in particular, could be eaten without affecting the face by sympathy.

It may be interesting for practical men to be informed, that all the above symptoms disappear almost at once on ascending the hill-land of Peru. In the interior the digestion becomes vigorous, the bowels natural, the irritability of the mucous membranes of the intestinal passages subsides, and the tone of the system is increased by moderate cold and regular exercise.

A visit to the Sierra, or interior of the country, is also a sovereign remedy for the most obstinate examples of hypochondriasis. In this gloomy infirmity the bracing air of the mountains, the excitement arising from the boldness and variety of the scenery, the feeling of danger by which a nervous person is frequently affected on the route, and the necessity of personal exertions on such a journey, have all a most beneficial tendency.

During some of the late campaigns in Peru, the delicate-looking sons of the coast were seen to return from a life of temporary hardihood among the hills, with all the characters of robust and vigorous health. By a change of climate, which can be easily effected in a few days, the most important and striking changes, physical as well as moral, may be accomplished; the weak may become strong, and the timid bold, as I have known in some most deplorable cases of hypochondriasis.

*Xaqueca.*—A kind of severe periodical headach, which is vulgarly called *Xaqueca*, is exceedingly frequent among the Lima women; and great numbers affected with it may be met with daily who have patches of some sort stuck on their temples by way of remedy. The xaqueca is commonly symptomatic of chronic disorder of the digestive functions, and it attacks with frequency women of a nervous habit of body, who are subject to congestion in the internal vessels of the abdomen.

When this distressing headach is connected with want of tone and vigour in the general system, or with dyspeptic symptoms of a nervous rather than inflammatory nature, decided advantage may be derived from keeping the bowels open, and giving for a longer or shorter time the carbonate of iron, which, of all tonics, I have found the best in this class of cases. But when connected with frequent indigestion and chronic visceral obstructions, I have seen the xaqueca relieved by a profuse and spontaneous hæmorrhoidal discharge, or by a deobstruent course of medicine calculated to correct the existing structural derangement.

*Vomiting.*—Of the various painful symptoms connected with deranged digestion, vomiting is one of the most prominent among the Limenese. It is sometimes attendant upon a highly irritable or chronically inflamed state of the mucous membrane of the stomach or intestines: sometimes it arises from mental emotion, or diseases in remote parts, which only affect the stomach sympathetically. At one time this symptomatic, but frequent and distressing affection, is kept up by structural, at another time by functional disease.

In order to illustrate its mode of appearance, and the sources from which it arises, I subjoin a few examples from my diary.

1. A young man in good health, having overheated himself by walking on a warm summer's morning, quenched his thirst by drinking iced-water. He had scarcely advanced two squares, or two hundred yards, from the place where he drank the water—that is, from the Palace Square to the Calle de San Jose,—when he was seized with giddiness, followed by violent headach, with redness of the eyes, and incapacity to stand or even sit up. He felt quite sick at stomach, and vomited the food which he had taken two or three hours before. He now drank warm water freely to favour the action of vomiting, by which he was so entirely relieved, that soon afterwards he made a good dinner.

2. A young girl, who had hardly attained maturity of form, was married to a young man who followed the lucrative occupation of baker in Lima; and, to indulge her in her childish inclinations, he allowed her to eat what they call *Antojos*, or fancy dishes. One evening she regaled herself with sweatmeats and *mani*, or ground nuts, until she became what they call *empachada*, or surfeited. Shortly afterwards, being excited to anger, she was seized with pain in the belly, some fever, and vomiting of green and yellow bile. No aliment except arrow-root or panada would remain on the stomach. *Caldo*, which is the name for mutton, chicken, or beef-tea, &c., especially disagreed with her, and was vomited as soon as it was taken. Upon the administration of an enema of milk and water, the vomiting ceased, and she speedily recovered.

3. In warm weather, a young woman, shortly after rising at her usual hour in the morning, and apparently in good health, was seized with bilious vomiting, which was induced by no assignable cause, and she also complained of great oppression or fatiga at stomach. She requested the aid of a physician, who prescribed a little gum-water with ice, to be taken every half hour. But, not being relieved by this, she consulted another, who ordered her one grain of opium in a bolus of conserve of roses. By this means the violent vomiting was restrained, and the cure was completed by iced-rice water.

4. A stout young man was suddenly seized with indigestion and vomiting after violent exercise. The vomiting was most distressing and almost incessant, attended with a convulsive agitation of the whole frame. Various external applications were resorted to in vain; nor did ice-water and effervescing draughts afford the least relief. Æther with laudanum in large doses proved equally unavailing; but all the symptoms very quickly yielded to a scruple dose of calomel, combined with one grain of opium.

5. A robust young man went to bed at his usual hour, and in his usual good health, in the warm month of February, after having drank an hour or two previously, a cooling beverage, which consisted of lemon-juice, water and syrup, with ice. He awoke at midnight, and had a sudden attack of vomiting; and afterwards, for many hours, the vomiting was attended with excruciating pain at the stomach and navel. The vomiting occurred every five or ten minutes; each attack being followed by an interval of rest, accompanied with a feeling of exhaustion; after which the pain returned, and the vomiting was renewed. The face was flushed; the surface was covered with general and incessant perspiration; the pulse was full and frequent; and the tongue clean and natural. The patient could not say whether or not on the first invasion he had rejected any undigested food; but he expressed his surprise that what he had vomited was neither bitter nor disagreeable to the taste.

I was called to see him about ten o'clock in the forenoon, when I learnt the facts of his case now mentioned. The symptoms had not yet much abated in their violence; and, under the circumstances, I thought it advisable to give him a dose of eighteen grains of calomel, mixed with a little gum arabic, which he took in common syrup, washed down with a few mouthfuls of plain water. Two hours after he drank a glassful of linseed water.

From the time he took the calomel the vomiting entirely ceased, and hardly any pain or disturbance in the bowels remained. The patent then became tranquil, and slept most of the afternoon. His bowels were not opened until the following morning, when he was quite recovered, and voided a moderate, consistent, and dark-coloured stool.

6. In cases of chronic gastritis, attended with frequent retching and vomiting of every thing taken by the mouth, I have known ice, which the patients chew with avidity, afford more relief than any other remedy, enabling the stomach to retain a spoonful or two of panada seasoned with some vegetable acid.

For diseases of this nature, not uncommon on the coast, cupping is less frequently resorted to in Lima than the interests of the sick require, in a place where leeches are extremely scarce. Blisters, sinapisms, embrocations, cataplasms, warm baths, anodynes, and clysters in abundance, are usually applied in native practice, and frequently without much benefit.

When all such ordinary resources have proved fruitless, it is sometimes discovered that the gastric disorder is kept up by sympathy between the stomach and some other organ in an unhealthy state; for example, an indurated and enlarged ovary, or uterus in a state of inflammation more or less acute. I well remember an instance of this latter nature, which was pronounced to be hopeless, and in which the patient was piously consigned, by a well-known native practitioner, to the care of the priest and confessor. But from this master's unpalatable opinion she appealed to a consultation at which I attended. She was now directed to take small doses of calomel in conjunction with opium, morning and evening, aided by mercurial inunction, until the salivary organs should become mildly affected. When this object was attained, the irritability of the stomach was so far removed that drink and farinaceous food could be comfortably retained, and by diluents and laxatives the cure was in a great measure completed, and the woman restored to the enjoyment of health, though the uterine enlargement was not entirely removed.

7. A young lady was confined to bed for three months, affected with obstinate costiveness and almost continual vomiting of green vitiated bile. Her complaint had originated in winter weather, and appeared in form of what she termed a bilious colic, which was soon followed, according to the statement, by symptoms of general inflammation in the bowels. These inflammatory symptoms had afterwards disappeared, but the costiveness and vomiting continued. Frequent warm baths, as well as opiates and iced-drinks, proved unserviceable, and nothing was found to relieve her. The case grew tedious. Many doctors were consulted, and at length it came to my turn to be called. I recommended the application of a large sinapism to the abdomen, to be followed by an enema, consisting of one drachm of ipecacuan powder in form of infusion, being administered, powder and all, with an addition of two tablespoonfuls of olive oil, and one of molasses. This compound opened the bowels freely, carrying off thin, yellow, and copious feculent motions; and the complete solution of a painful and protracted malady was the consequence. The patient rapidly convalesced; but was very attentive to the quantity and quality of her diet for some time.

I may remark that in this case the enema employed appears to have stopped the vomiting chiefly by the revulsion it occasioned, just as at other times we see that an emetic dose of the same powder suspends or removes a troublesome diarrhœa.

8. I have met with some instances of periodical vomiting, arising at a certain stage of the process of digestion, and attended with or preceded by, pain more or less acute.

In such cases the appetite may be good enough; but a few hours after food is taken, that is in about from three to five hours, there comes on a severe colic, followed by vomiting; and at length a portion of fetid and half-digested matter is brought up with much effort; giving the individual affected relief till after eating again, when he has again to go through the same course of suffering.

I particularly remember two examples of this kind of severe gastrodynia. One took place in a young man a good deal addicted to the use of ardent spirits, and the other in an elderly person, of a choleric temperament, and much exposed to the sun in his daily agricultural pursuits. In neither case could any abdominal tumour or induration be detected by external examination. Both subjects consulted various physicians, and were led to use many different drugs, finding none that was efficient; and both, at last, after long illness, were cured by bathing in the sea during the summer season.

9. It may be curious to mention that a white woman, residing in Lima, and in the middle ranks of life, was accustomed to have two dinners prepared for herself daily. The first dinner was a sumptuous one, abounding in all the rich varieties of the season. Of this she partook very freely as a luxury; but shortly afterwards, this preliminary repast was wholly rejected by the aid of draughts of warm water; and, when the stomach had recovered a state of repose, she eat her second or plain dinner, which served to nourish and support her very corpulent frame.

Such a debility of stomach as the following case exhibits is not common.

10. A foreigner, who had resided in Lima for many years, complained of weakness at stomach, indicated by vomiting his food, with some slight uneasiness, very soon after taking it. This gentleman could not perspire, and the radical disease under which he suffered was supposed to be seated in the liver. Though he passed the winter in Chorillos on account of its milder climate, and took sufficient daily exercise in riding to and from Lima, yet his skin continued dry and stomach weak. When in good health he perspired freely, and he therefore thought that a voyage to Guayaquil might do him good, by restoring the naturally perspirable state of the surface. He embarked, and before he got to his destination, the skin became relaxed, and he felt better. So rapid was his recovery that, as he avers, on the very first day of his arrival in Guayaquil, he eat three hundred of the small oysters which adhere to the leaves of the mangroves that dip in the river of this city. After this feat he had no more ailment of any kind; the functions of the skin, and also of the stomach and intestines, became perfectly healthy and natural.—*Ed. Med. and Surg. Journal.*

---

*Three remarkable Cases of Disease of the Pharynx and Œsophagus.* By Professor ROKITANSKY, of Vienna.

1. *Dilatation of the œsophagus.*—K. J., ætat. twenty-four, a servant, suffered for nine years from vomiting, which especially occurred after eating indigestible food. In the winter of 1833, he came to the hospital after four days' illness, the apparent consequence of having got drunk on beer. He complained of nausea, desire to vomit, faintness, hiccup, constipation, and thirst: the features were fallen; the eyes sunk; their lids livid; the skin dry; the temperature less than natural; the pulse very small and frequent; the tongue covered with a thick white fur; the abdomen large, rather hard, and somewhat sensitive in the epigastrium; the breathing short and quick, with a dry cough. Tartar emetic was given, and in the evening produced diarrhœa and vomiting of very stinking, pulpy food, with great relief to the patient, but the cough and hiccup had increased. Oxyde of bismuth was given in half-grain doses with the emetic.

Next day there was copious vomiting of a brownish bloody material, with frequent hiccup, ringing in the ears, and cough, and the pulse was scarcely to be felt. On the following days, also, after leaving off the tartar emetic, the vomiting continued in a less degree. On the ninth day of the disease, however, it ceased; but the diarrhœa, hiccup, and coldness of the extremities, continued, and the general debility was increased. On the tenth day the diarrhœa ceased, and the body became uniformly and unusually warm; but, on the twelfth day, the diarrhœa recurred more violently than before; the abdomen became painful all over, and the temperature again fell below its right standard. On the 13th the patient died, in an extreme degree of exhaustion.

At the post-mortem examination, the contents of the chest were found moderately healthy. The liver was brittle and full of blood, and consisted of coarsely granular, clear- and dark-brown substances. The gall-bladder was distended by thick, blackish-brown bile, The spleen was shrivelled, dense, and purple-red; the pancreas pale-reddish, full of blood. The solar ganglion was purple-red; its outer substance beset by numerous punctiform extravasations of blood; the neighbouring venous plexuses turgid. The intestines were partially contracted; the jejunum contained a brownish mucus, the lower part of the ilium a dark-red mucous and bloody pulp, and the large intestine a yellowish soft fæces. The mucous membrane of the intestines was every where swollen: in the jejunum it was pale; in the lower part of the ilium dark red, with congested veins, and enlarged mucous glands. The kidneys were small, and full of blood.

The stomach contained a considerable quantity of blackish-brown fluid, mixed with chocolate-coloured lumps; its mucous membrane was swollen. The opening of the œsophagus was narrow, and formed a prominent folded ring in the cavity of the stomach, like the vaginal portion of the uterus. Above it, the œsophagus, up to the pharynx, was found dilated into a spindle-shaped pouch, which, at its middle, would have received a moderate-sized man's arm. It was filled, like a sausage, with a blackish-brown soft mass of small pieces, which formed a consistent cylinder covered externally by a layer of white mucus. The membranes of the œsophagus were generally thickened, especially the muscular coat, whose internal and circular layer of fibres was nearly a line thick. The muscular fibres were pale; the mucous membrane was thickened, and its tissue was traversed by very dilated but empty blood-vessels. From about an inch above the cordia, to the upper third of the canal, there were numerous ulcers, as large as lentils and silver *groschen*; they were round, with sharp-cut edges, and were here and there grouped or coalescing; their bases were formed within by the exposed muscular coat, or by a blackish-brown adherent slough, in whose tissue one could see numerous vessels, with their orifices closed by coagulum.

2. *Diverticulum on the pharynx.*—A. A. æt. sixty-six, a journeyman-tapemaker, of a tolerably robust form. On his admission into the hospital in August, 1837, said that he had been healthy up to his seventeenth year, with the exception of the common diseases of childhood, and, especially, repeated swellings of the cervical glands: in that year he observed the formation of a tumour in the right side of the neck, about three fingers' breadth below the angle of the lower jaw; it was not painful, but it gradually acquired the size of a pigeon's egg, and after three years it appeared to him to have extended a good deal forwards; at that time, *i. e.* in his twentieth year, he found a difficulty in swallowing, by being almost constantly annoyed by the sensation of a foreign body sticking in the upper third of the œsophagus, from which he was sometimes relieved at night, when he lay on his back, by vomiting some of his food. He especially recollected having once suffered thus for three days, till the vomiting of a small quantity of perfectly undigested food, which he had eaten three days before, immediately relieved him.

This condition remained almost stationary for forty-three years, but for the last three years the difficulty of swallowing had greatly increased: he could only take fluids, and even these at last caused him the same forcing agonizing sensation: the vomiting occurred more frequently, and was accompanied by rapid emaciation. Two months ago, moreover, he had had an inflammation of the lungs.

On examination, there was found on the right side of the neck a firmly elastic tumour, nearly as big as a man's fist, reaching from the region of the larynx obliquely inwards and downwards to the right sterno-clavicular articulation, and even passing behind it into the cavity of the chest. In this tumour there was discovered the right lobe of the thyroid gland, hardened into a fibrous cyst, which the patient accused as the cause of his wretched condition.

On the left side of the neck it was noticed that the œsophagus was blown out every time the patient drank, and that it formed a swelling as big as a pigeon's egg, which always disappeared directly after he vomited what he had drunk. Vomiting of the fluid just taken was produced by pressure on this swelling, as well as by lying horizontally on the back.

For three days, he said, he had vomited nearly the whole of the food that he had taken; every attempt to introduce an instrument into the œsophagus was useless; it always came to a considerable constriction at one and the same spot, and beyond this it could never be passed. The patient was kept alive for eighteen days by nutritious enemata and baths.

In the organs of the chest and abdomen nothing remarkable was found on the post-mortem examination. The left lobe of the thyroid gland was small and flaccid; the right was converted into a fibro-cartilaginous thickly-walled sac, as large as a goose's egg, containing a turbid puriform fluid, fibrinous coagula, and some bony concretions. This sac descended behind the right sterno-clavicular articulation into the cavity of the chest, receiving at its lower part a slight impression from the right clavicle, and flattening, in some degree, the trachea. The mucous membrane of the fauces and pharynx was thickened; that of the epiglottis and ary-epiglottidean folds was œdematous. At the inferior constrictor of the pharynx the mucous membrane was prolonged through the lowest horizontal fibres of that muscle into a diverticulum more than two inches long, and at least as wide as the pharynx, which was held by the cellular envelope of the œsophagus in such a position that the canal of the pharynx led straight into its cavity instead of into the œsophagus; so that whenever one tried to pass a finger or a sound into the latter it always passed into the pouch. It consisted, besides some few bundles of pale muscular fibres, of a thick layer of cellular tissue and a thickened mucous membrane, polished internally like a serous membrane, which was straightened at the bottom of the hernial protrusion by an irregular reddish deposit like that of lymph in serous cavities. Just below the mouth of the diverticulum the œsophagus was very much narrowed, and its canal had only the form of a small transverse fissure; it continued thus also in its further course: its muscular coat was thick; its mucous membrane was constricted in thick longitudinal folds, and the veins ramifying in the subcutaneous cellular tissue were varicose.

3. *Fibrous Polypus of the Œsophagus.*—F. M., æt. 48, a day-labourer, of tolerably robust form, was taken into the hospital in February 1834. According to his account he had suffered some years ago from ague, and had thence become dropsical. About two months ago he first perceived a pain in the fauces, with difficulty in swallowing; the pain subsequently descended into the dorsal region, and was accompanied by cough and feverishness. In the hospital he had a cough, which was especially troublesome at night, and afterwards cachexia, palpitation of the heart, anasarca, especially of the fore and upper extremities, and constipation, were the most prominent symptoms. The difficulty of swallowing seemed to have disappeared, for the patient, who, on his first admission, described it as but slight, at last ceased to feel it at all, and had a good appetite up to the 19th of May, when dyspepsia came on. After that time the dropsy rapidly increased, and he died May 27.

The stomach, at the post-mortem examination, was found moderately distended, and it contained, besides gas, a grayish-white mucous fluid. Its mucous membrane was very much thickened, and covered with a gray, jelly-like mucus. The œsophagus was distended by a substance firmly seated in it, which, on opening its canal, was found to be a polypus, hanging from the pharynx, and reaching down to about two and a half inches above the cardia. It proceeded from the anterior wall of the pharynx, just below the posterior part of the glottis, where it was attached by a flattened round stem, about four lines thick, composed of the submucous cellular tissue, and it hung down in the œsophagus, covered by a thin mucous membrane. It was altogether seven and a half inches long, and it increased in size from above downwards, so that its slightly lobulated and knobbed lower end was two and a half inches in diameter. The œsophagus was closely contracted around its middle; externally, and especially towards its free end, it was bluish red; the mucous membrane covering it was injected, and covered by a puriform exudation; its substance was reddish yellow, very flaccid, soft, elastic, and composed of a fibro-cellular tissue. Just above the cardia there was an ulcer of the mucous membrane of the œsophagus.—*Lon. Med. Gaz., from Medicinische Jahrbucher des K. K. österreichischen Staates; Bd. xxi. St. ii.*

*Philosophical and Experimental Researches into the true and Primary Action of the Secale Cornutum as a Medicine.* By Dr. C. T. De Gravina.—The first experiments were made upon five full-grown healthy sparrows, to three of which eight whole grains of the ergot were given, and to the two others six grains a piece, powdered and formed into pills by means of syrup. The two latter were the first to feel the effects of the drug, and then those which had taken the whole grains; but both exhibited the same phenomena. They first ceased to chirp and flutter when the cage was approached; they then remained immovable, and could with difficulty support themselves; next, they erected their feathers, which they shook from time to time, as though to free themselves from something that oppressed them and irritated their skins; then they violently shook their heads from side to side, opening and shaking their beaks, with an effort to vomit, which after a time took place, when they threw up fragments of the pills, or whole grains of ergot, soaked in a clear and frothy fluid like saliva. The muscular weakness increased to perfect immobility unless they were much teazed, when they removed a few steps from the spot and relapsed into their former state; and when thrown into the air they soon fell to the ground. The respiration and pulse became slower, and with the animal heat decreased gradually till their death, which took place in six or seven hours after swallowing the ergot. Examined immediately after death, the viscera presented no inflammatory morbid appearances. The lungs were healthy, a little congested. The right ventricle, auricle, and the venæ cavæ were distended with blood, and the spleen was much congested. The brain presented no morbid changes, but the muscles were fragile and contained black blood. Two guinea-pigs were the subject of the next experiment, a male and a female, the latter ten days advanced in her second pregnancy. Twenty-four grains of ergot, powdered, and made into a paste with flour and water, were given to each daily, before their other food, and the dose was increased each day by the addition of twelve grains, until it amounted to seventy-two grains. In about an hour after each dose they became immovable and indifferent to any disturbance, but re-composed themselves immediately. The heart beat very slowly, and they refused every kind of food. Two or three hours were passed in this state, and then they returned to their former liveliness; but the effects of the ergot were more distinct and permanent in proportion as the dose was augmented. The female, though pregnant, exhibited no increase in size during the eight days that she took the ergot, nor indeed after it had ceased to be given; so that Dr. De Gravina began to doubt the fact of her pregnancy, especially as the time for her bringing forth had now passed; and although her belly might have been rather tumid yet that was attributed to fat, as she took a great deal of food, and had been confined from the commencement of the experiment in a pen by herself. However, twelve days after the usual period she produced three very lively young ones, one of which was blind of the right eye.

Wishing to try the effects of the ergot upon a healthy person, Dr. De Gravina swallowed twenty-four grains in powder, which, in three quarters of an hour produced a sensation of weight in the epigastrium, which, slight at first, became in a short time very painful. This was followed by nausea and eructations having the odour of the ergot, and terminating in attempts to vomit. His face became very pale and the skin cold, particularly that of the face; there was great oppression in the head, with incapacity for any mental exertion, apathy, and a feeling of complete prostration and vertigo on walking across the room. The pulse, which before the experiment was 65, fell to 54, small and slow; and the respirations decreased from 18 to 13 per minute. There was a complete disgust for food, with an occasional feeling of coldness at the stomach that thence pervaded the whole frame. All these symptoms were instantly removed, and a voracious appetite was restored, by simply taking a glass of strong wine.

Having thus, he says, ascertained that wine was an antidote to the effects of ergot, Dr. De Gravina resolved to try whether opium and mace would have an equal power to prevent the sedative action of the drug when administered with it; but, in order to learn what would be the effect of the opium and mace given separately, he previously instituted the following experiments:

A grain of opium was given to a sparrow just caught, and a grain and a half to another. In half an hour the former became exceedingly lively, chirping, fluttering, and turning his head in every direction in a most excited manner for twenty minutes, when he fell asleep. His breathing was frequent, and his heart beat most rapidly, and the heat of his body was much increased. Roused by a kind of convulsion, he vomited a fluid like saliva, in which a little opium was dissolved, and then recomposed himself to sleep, during which universal tetanic spasms came on, followed by paralysis with atonic spasms of the right side. The tetanus became more frequent and violent, and in one attack he vomited a larger quantity of similar matter as before; the attacks then became gradually less violent and frequent; he fell again into a deep sleep, from which awaking by degrees he returned to his previous state of health. The second sparrow exhibited the same symptoms, but in a more violent degree, and having been prevented from vomiting by a ligature round the neck, the tetanic spasms continued incessantly to his death. The cerebro-spinal axis was vividly injected in every part, and the digestive tube was much the same; the lungs were gorged with florid blood, and the muscles were rigid and tore with difficulty, nor were they at all injected with dark blood as in the experiments with the ergot.

Having now established (as he imagined) the action of both substances singly, Dr. De Gravina gave another sparrow half a grain of opium with three grains of ergot in a pill. In three quarters of an hour the effects of the opium appeared as before, with great liveliness of motion, but instead of passing into a state of stupor, the animal became depressed and exhibited all the signs of poisoning by ergot. In this state he gave it four drops of laudanum, which instantly removed all the symptoms. In repetitions of the last experiment, the opium always counteracted the effects of the ergot, with or without exhibiting its own, according to the proportion it bore to the dose of ergot.

The effects of mace were much the same as the early symptoms produced by opium, but in an extreme degree; and after the animals had remained about half an hour in this state, a slight opisthotonos appeared, increasing rapidly till near the death of the birds, when they began a progressive movement in a straight line, turning neither to left nor right, but continuing to push against any obstacle that opposed itself insurmountably to their progress, and moving their legs as though they were swimming. A strong bronchial rattle with panting came on before death, which occurred about four hours after they had taken the mace. Immediate dissection disclosed the intestinal tube highly injected; the lungs hard, hepatized, and engorged with very florid blood; a bloody serous fluid filled the trachea; the parietes of the heart were redder than usual; the venous blood was of a bright red and gave out a strong smell of nutmeg; the vessels of the brain and spinal cord were very conspicuous and of a most briliant colour. These experiments were repeated with various doses of the mace, but with the same results; and also with mace combined with ergot, when each in turn produced its peculiar effects, but with a modified intensity, as resulted from the combination of opium and ergot.

Cicuta given to sparrows produced direct depression and stupor without any previous excitement, and combined with ergot, death without a struggle. The morbid appearances were the same as in poisoning with pure ergot.

Dr. De Gravina next cites a case of irritable uterus, with diseased ovary, first relieved by cicuta, and in a second attack by ergot, to prove the identity in the mode of action of these remedies; and several cases of hæmoptysis and active inflammations treated with bleeding, ergot, ice, and digitalis, in order to establish the same point in regard to them.

From these experiments and cases, Dr. De Gravina concludes that ergot, like cicuta and digitalis, is a direct sedative, and that it is as directly opposed in its action to opium, mace, and wine. Hence he considers it a good antiphlogistic remedy, and well calculated to lower the vital powers.

With regard to its action on the uterus, he cites several cases from which he concludes that the condition of the uterus that renders it liable to be stimulated to contraction upon its contents, is when there is a degree of morbid vigour in the organ, and not when it is in a state of real debility, for then the ergot is worse than useless, as it induces general depression.

[The reader will at once perceive the imperfections in the mode of experimenting, and the chasms and weaks points in the chain of reasoning in the foregoing paper. Still the experiments have some value as single facts; and they deserve more consideration from their results coinciding with those of Mr. Wright, (Edin. Med. and Surg. Journal, No. 142,) more particularly in the fact of the power of ergot in prolonging gestation in the guinea-pig and rabbit, when administered for a considerable period.]—*Brit. and For. Med. Rev. from Annali Universali di Medicina. Ottobre*, 1839.

---

*On Poisoning by Antimonial Vapours.* By Dr. Lohmeier, of Schönebeck.—The subjects of this peculiar affection were four in number. The history of the first is as follows:

D. W. E. F. was affected, in 1838, with tightness in the chest and slight pain of the head. The former symptom increased to pain, and at last to severe stiches passing transversely through the chest to the shoulders and the back, to which there was also added a severe, dry, and painful cough. With these symptoms the pain of the head, which was at first but slight, acquired an agonizing severity, with stabbing and burning sensations, especially at the back of the head and the neck, and with swelling of the cervical glands. The dry, painful cough at last produced a difficult expectoration, while in respiration a ronchus and sibilus were constantly audible. At night a tormenting restlessness kept the patient awake; and this soon passed into complete watchfulness; or, if he fortunately had a short sleep, he fell into a distressing colliquative sweat, which was followed by great faintness. The appetite diminished from the very commencement of the illness, and diarrhœa came on with griping pains in the abdomen. The diarrhœa was troublesome and frequent; the food was evacuated undigested soon after eating; and the abdomen was enlarged and tense. There were pain and difficulty in making water, and at last the passage of the urine was always accompanied by strangury and pain at the neck of the bladder, and burning sensation in the urethra, from which a few drops of a liquid mucus sometimes flowed. Pains in the testacles were felt, and there was a loss of the desire for the exercise of the sensual propensity, which went on to actual impotence; and the patient observed a remarkable shrinking of the penis and an actual diminution in the size of the testicles.

The cases of the three other patients are so nearly similar to the above that they need not be detailed. After describing them Dr. Lohmeier proceeds: These histories had so much similarity that one and the same cause of disease in all the patients might fairly be anticipated; and it was the opinion of one of them who, like the others and some more who they said were similarly affected, had been occupied at the time of his illness in making antimonial preparations on a large scale, that the vapours given off during the process were the cause of his disorder. The idea, however, was not followed out till the same patient having almost entirely recovered from his first attack, but being still annoyed by pains, found them suddenly break out again into more severe symptoms on being exposed to the vapours developed in preparing a large quantity of tartar-emetic.

The author was now induced to inquire more accurately into the origin of these affections, which both by their history and their symptoms appeared so strongly indicative of the influence of some irritating poison. He found that one of the patients, D. W. F., at the time of his illness had been engaged for fourteen days successively in the manufacture of metallic antimony, by melting algorath-powder with fluxes composed of salts of potash and soda, at first in crucibles and afterwards in a small blast furnace. He melted every day about a hundred weight of metal, which was done in from four to six meltings, according to circumstances; and he had to test it during the time, and looked after the stirring of it himself. During this process vapours of antimonious and antimonic acids, and of oxyde of antimony are given off, from those substances being mixed with each quantity of the algaroth-powder that is thrown into the furnace.

G. H., another of the patients, had the management of solutions of antimony in hydrochloric acid in an open cast-iron cauldron, in which vapours of hydrochlorate of antimony (butter of antimony) were formed.

J. F., the third patient, was engaged in the same work with the last; and K. J., the fourth, was constantly occupied in preparing first the *vitrum antimonii*, and afterwards the artificial sulphuret, which is made by melting together sulphur and metallic antimony; in both of which processes vapours are given off containing antimonious and antimonic acids and oxyde of antimony.

There could, therefore, be no doubt that the peculiar and severe symptoms produced in these cases were the result of the absorption of the different compounds of antimony in the form of vapour. The remainder of the very interesting paper is occupied by the author's speculations respecting their mode of reception into, and action upon, the system; and by his suggestions for the prevention and the cure of the injurious effects to which they give rise. For the latter purpose he recommends local bleedings from the neighbourhood of the parts chiefly affected, and the concurrent administration of tonics, especially bark, which he presumes must exercise a chemical influence on the poison. For the prevention of poisoning no other than the usual means, such as powerful draughts of air for the removal of the vapours as fast as they are given off, seem to be necessary.—*Ibid. from Casper's Wochenschrift. April und Mai*, 1840.

---

*The Rest-harrow (Ononis Spinosi, or O. Arvensis,) a Remedy for Rheumatism.* By Dr. Ascherson, of Berlin.—Of this, as of many other remedies for chronic rheumatism, little more can be said than that a respectable author adds his testimony to the correctness of popular belief. Dr. Ascherson first heard of the virtues of rest-harrow (a common weed in this country as well as in Germany) from a washerwoman, and being convinced that it cured her, he tried it in several other cases and found it surprisingly beneficial. It was not invariably successful, but it never did harm, and cured many cases that had long resisted other means. The form of administration is a concentrated decoction of the fresh herb with its roots, or of the roots and stems dried; and a quart of the decoction must be taken daily. Its immediate effect is powerfully diuretic.—*Ibid. from Caspar's Wochenschrift. June* 6, 1840.

---

*Experimental Researches on the Functions of the Encephalon, considered in relation to Sensation, Station, and Progression.* By M. Monat.—This is a long report to the Royal Academy, in which the author, after proving the insensibility of the brain, endeavours to point out the functions of its different portions. In this, however, he has but partially succeeded, and the Committee, MM. Ribes, Blandin, Amussat, and Bouillaud, come to the following conclusions: 1, That the three great nervous centres, the spinal marrow, the cerebrum, and the cerebellum, have distinct and special functions; 2, that sensation, properly so called, and motion are under the immediate and direct influence of the spinal marrow, and have each particular nerves; 3, that the brain presides over the various manifestations of intelligence and volition; 4, that the cerebellum plays an important part over the functions of *Progression* and *Station.* As to the functions peculiar to particular parts of the nervous centres, much yet remains to be done for their determination.—*Ibid. from Bull. de l'Acad. Roy. de Méd. Juin* 17, 1840.

---

*Mode of discovering Adulteration of Essential Oils with Alcohol.* By M. Borsarelli.—M. Borsarelli introduces the essential oil into a cylindrical tube about one inch in diameter and four in length, closed at one end. The tube is to be about two-thirds filled with the oil. He then introduces small pieces of chloride of calcium, very dry and free from dust, closes the open end of the tube with a cork, and places it for four or five minutes in a water-bath heated to 100°, occasionally shaking it. The tube is then allowed to cool gradually, and if the oil contain any considerable quantity of alcohol the chloride is entirely dissolved, forming two distinct layers—the superior is the essential oil, the inferior the alcoholic solution of chloride of calcium. If the oil contain but a very small proportion of alcohol the calcareous chloride effloresces, loses its form, and becomes a white mass which adheres to the bottom of the tube. Finally, if the oil contain no alcohol, the chloride not only does not dissolve but retains its proper form.

The same process may be employed to discover the proportion of alcohol mixed with æther, remembering, however to use a longer tube, and not to cork it too tightly.—*Ibid. from Bulletin Géneral Thérapeutique.* 30 *Juin*, 1840.

---

THOUGHTS ON MEDICINE.

Callicrates is a medical system monger. He takes an idea, and attaches himself to it; broods over it, fecundates it, fashions and stretches it beyond all measure; and it sometimes becomes a fixed idea, a monomania. Let him once bestride the back of his systematic chimera, and he is ready to fight to the last extremity. Experience, facts, phenomena, observations, inductions, principles, however opposed to his system, do not trouble him in the least; he applies his magnifying-glass to them, and from that moment sees what he wants to see, and as he wishes to see it. "There is nothing," he says, with energy, "better demonstrated or more certain; a man must be very ignorant, or very dishonest, not to see as I do;" this is the pathognomonic symptom of his madness. After this every proof suits him, every argument is good. However little you press him, Callicrates will maintain that white is not white, that a stick has not two ends. He reminds one of that bold and subtle dialectician who had an answer for every thing. Some one said, "the north wind is blowing;" "by no means," he replied; "it is the south wind—*but it is going home.*"

—

When a student came to solicit the protection of Louis, the celebrated secretary of the Academy of Surgery, the latter never failed to ask, "Do you know anatomy?" "Yes, sir." "Well, then, describe the stool which stands before you." Louis was right, and used an excellent means of fathoming a man's understanding, and making out his future destiny; in this way he judged of the sagacity, the logic, and the methodic manner of the candidate. There is an infinity of things in our art which are like Louis's stool, and many persons would find it difficult to describe them well.—*Med. Gaz.*

